# Retroperitoneal endoscopic median arcuate ligament incision with interventional radiology: a case report and literature review

**DOI:** 10.1097/MS9.0000000000000243

**Published:** 2023-03-24

**Authors:** Shoryu Takayama, Satoru Takayama, Hisanori Kani, Akimitu Tanaka, Ken Ishikawa, Nobuyasu Yoshimoto

**Affiliations:** Departments of a Surgery; b Cardiology, Nagoya Tokushukai General Hospital, Kasugai-Shi, Aichi Prefecture, Japan

**Keywords:** interventional radiology, median arcuate ligament compression syndrome, pancreaticoduodenal artery aneurysm, retroperitoneal endoscopic surgery

## Abstract

**Introduction and Importance:**

Compression of the celiac artery (CA) associated with median arcuate ligament compression syndrome can result in aneurysms at the pancreaticoduodenal arcade. If the aneurysm ruptures, treatment with interventional radiology (IVR) is recommended. Subsequently, the median arcuate ligament (MAL) should be incised to prevent the recurrence of the aneurysm. Retroperitoneal endoscopic MAL incision reduces the risk of adhesive bowel obstruction. However, there is few surgical landmark for retroperitoneal MAL incision. We used IVR to detect CA for MAL incision.

**Case Presentation:**

A 44-year-old man presented to our hospital with complaints of abdominal pain and clouding of consciousness. Contrast-enhanced computed tomography of the abdomen showed contrast leakage from pancreaticoduodenal artery aneurysm, and the CA was compressed by MAL, leading to the diagnosis of pancreaticoduodenal artery aneurysm rupture associated with median arcuate ligament compression syndrome. IVR was performed to block the blood flow to the aneurysm. After 2 months from life-saving IVR, we performed retroperitoneal endoscopic MAL incision with IVR. The patient was discharged 8 days after surgery. Echocardiography and contrast-enhanced computed tomography 2 months after discharge confirmed that the compression and flow of the CA had improved.

**Clinical Discussion:**

In retroperitoneal endoscopic MAL incision, there has been few landmark to identify MAL and CA. Retroperitoneal procedure with IVR can identify MAL easily. This is a useful technique, and it is important to accumulate more cases to standardize the technique.

**Conclusion:**

Retroperitoneal endoscopic MAL incision with IVR has not been reported, this procedure can make it easier to detect MAL.

HIGHLIGHTS

- We report a case of retroperitoneal endoscopic median arcuate ligament (MAL) incision with interventional radiology (IVR).
- This procedure makes it easier to identify MAL and celiac artery (CA) in retroperitoneal approach.

## Introduction

This work has been reported as in line with the SCARE 2020 criteria^1^. Rupture of pancreatoduodenal artery (PDA) aneurysms associated with median arcuate ligament syndrome (MALS) has been reported in many cases^2^,^3^. When an aneurysm ruptures, treatment by IVR is recommended rather than surgery^4^. PDA aneurysms form when the compression of the CA by the MAL changes blood flow and pressure at the pancreaticoduodenal artery arcade. Therefore, a MAL incision is performed to release the stenosis of the CA as a radical operation. Laparotomy, laparoscopic surgery, and retroperitoneal endoscopic surgery have been reported^5^,^6^. It is a relatively rare syndrome, so treatment procedure is controversial. There have been reports of failed MAL incisions because of difficulty of identification of MAL^7^. Even in our own cases, CA identification has been difficult. In this case, we used IVR to identify the location of the CA and performed surgery to ensure MAL incision.

## Case presentation

**Figure 1:**
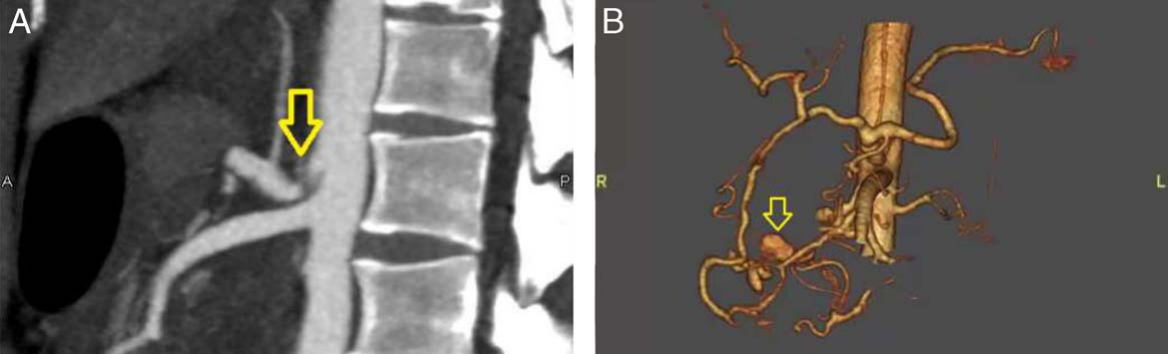
Pancreatoduodenal artery aneurysm due to median arcuate ligament syndrome. Contrast-enhanced computed tomography was performed. Blood flow from the superior mesenteric artery increases with compression of the celiac artery due to median arcuate ligament syndrome (A). This causes a change in hemodynamics within the pancreaticoduodenal arcade and forms an aneurysm (B).

**Figure 2:**
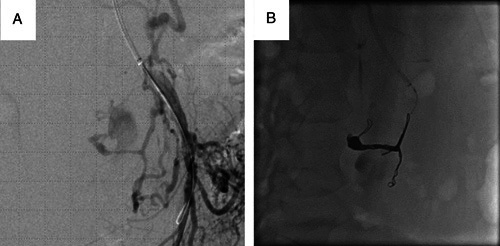
Interventional radiology for ruptured pancreatoduodenal artery aneurysm. Extravasation from the pancreaticoduodenal artery aneurysm, which was noted preoperatively, was observed (A). Coil embolization was performed and adequate hemostasis was achieved (B).

**Figure 3:**
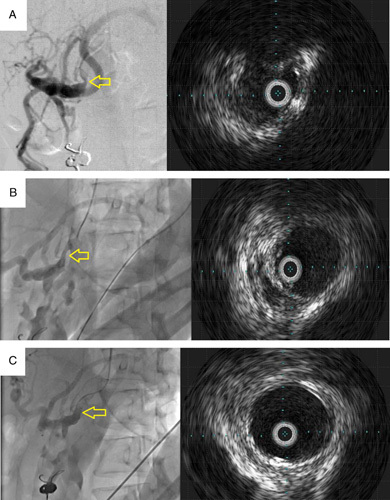
Interventional radiology and intravascular ultrasonography before and after incision of the median arcuate ligament. Interventional radiology and intravascular ultrasonography after coil embolization of pancreatoduodenal artery aneurysm (A). After coil embolization, the celiac artery was still compressed by the median arcuate ligament, and intravascular ultrasonography showed evidence of stenosis. After median arcuate ligament incision, interventional radiology and intravascular ultrasonography were performed again (B). The incision resulted in the dilation of the celiac artery and lumen of the vessel. The celiac artery was additionally balloon dilated. Interventional radiology and intravascular ultrasonography were performed after dilation (C). We determined that the hemodynamic abnormalities associated with median arcuate ligament had improved after confirming adequate celiac artery dilation.

**Figure 4:**
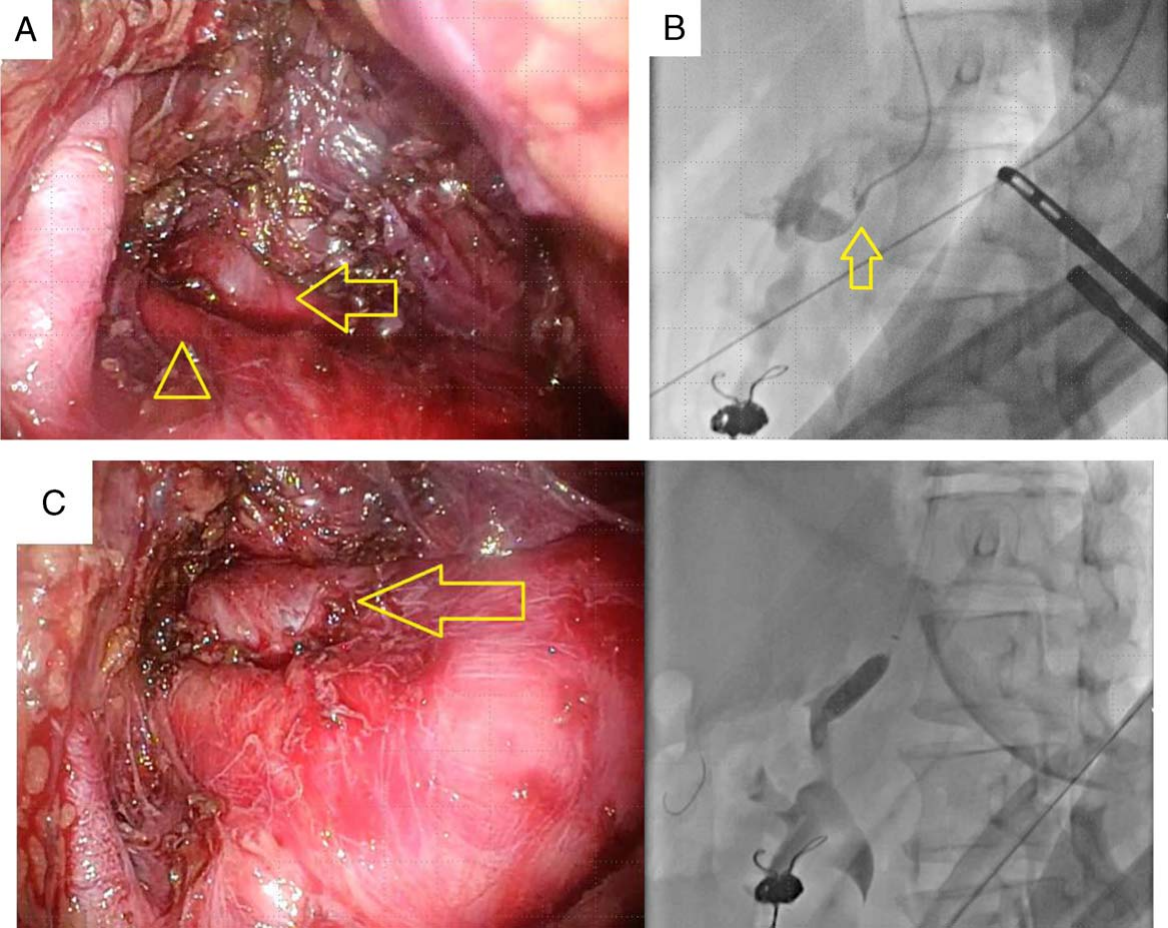
Laparoscopic median arcuate ligament incision via retroperitoneal approach. The median arcuate ligament incision was performed with interventional radiology. The superior mesenteric artery (arrowhead in A) and celiac artery (arrow in A) were identified. Interventional radiology was used to identify the celiac artery, and laparoscopic procedures were performed with the catheter engaged into the celiac artery (B). Thanks to this procedure, the median arcuate ligament nearby the celiac artery could be easily identified and incised. After incision, the celiac artery was balloon dilated (C). Since balloon dilation was performed while observing through the laparoscope, we were able to confirm the expansion of the celiac artery in real time.

A 44-year-old man with no specific medical history presented to his previous physician with a chief complaint of abdominal pain. He was transferred to our hospital on suspicion of ruptured abdominal aortic aneurysm. Two liters of transfusion was administered to maintain blood pressure. When he arrived at our emergency room, his blood pressure was 75/50 mmHg and heart rate was 110/min. His consciousness was clouded. Physical examination revealed tenderness in the pericardial area. Contrast-enhanced computed tomography (CT) of the abdomen showed contrast leakage from the PDA mass, and the CA was compressed by MAL, leading to the diagnosis of PDA aneurysm rupture associated with MALS (Fig. 1). IVR was performed to block the blood flow to the aneurysm (Fig. 2). There was no evidence of extravasation from the aneurysm after hemostasis. Abdominal distention due to intra-abdominal bleeding was prominent, and intravesical pressure was measured at 30 mmHg. There was concern about abdominal compartment syndrome, but since the patient’s hemodynamics was stable and there was no evidence of organ damage, laparotomy to decompress the abdomen was not performed. After IVR embolization, the patient was still intubated and admitted to the ICU to perform decompressive laparotomy immediately when it was needed. After admitting to the ICU, continuous aspiration was performed from the nasogastric tube. Two hours after the first IVR, his intravesical pressure had dropped to 10 mmHg. The patient was extubated the day after the first IVR. Intravesical pressure had normalized. Abdominal compartment syndrome did not occur. The patient’s condition stabilized and he was discharged on the ninth day of hospitalization. Therefore, the patient was admitted 2 months after treatment with IVR (Fig. 3) and underwent retroperitoneal endoscopic MAL incision (Fig. 4). There were no complications and the patient was discharged 8 days after surgery. Echocardiography and contrast-enhanced CT 2 months after discharge confirmed that the compression and flow of the CA had improved.

## Treatment procedure

### Transarterial embolization for pancreaticoduodenal artery aneurysm rupture

The plan was to embolize the aneurysm with IVR. The CA was to be approached from the upper extremity and the superior mesenteric artery (SMA) from the lower extremity. An arterial line was placed in the right radial artery. A 6 Fr sheath was inserted into the left brachial artery. A 7 Fr sheath was inserted into the right femoral artery. The approach was started from the upper extremity to search for the CA, but only the SMA could be approached. Contrast was injected from the SMA to identify the site of bleeding (Fig. 2A). The CA was enhanced retrograde from the SMA, and it was determined that the CA was completely occluded by the MAL, so the approach from the CA was abandoned and embolization of the aneurysm from the SMA was attempted. Digital subtraction angiography from the SMA was performed to create a road map. Then coil embolization for PDA aneurysm was completely performed (Fig. 2B). Digital subtraction angiography was performed again to confirm that there was no extravasation. The hemostasis was completed.

### Retroperitoneal endoscopic median arcuate ligament incision with interventional radiology

IVR performed first in the supine position. We expected that the hemodynamics of the pancreaticoduodenal arcade would be altered by embolization of the aneurysm. Unlike first-time IVR, the CA was not obstructed completely and could be engaged. However, it was still highly stenotic. Intravascular ultrasonography (IVUS) was performed to evaluate the severity of the stenosis (Fig. 3A). Then the location of the CA was ascertained using the vertebral body as a marker. The catheter was removed because heparin had to be administered with the catheter in place. The patient was placed in the right lateral recumbent position, and laparoscopic surgery was started. A skin incision was made in the left lateral abdomen to make a retroperitoneal space. The retroperitoneal space was made with a balloon for dissection. Three-port retroperitoneal endoscopic surgery was started, and we attempted to identify the CA with the landmark of vertebral level, but we could not identify. Therefore, we decided to perform laparoscopic surgery with IVR, allowing for a bleeding tendency with heparin. The surgical position was left side of the abdomen tilted at 15°. The SMA and CA were identified with IVR (Fig. 4B), and the MAL was incised (Fig. 4A). At this point, the vessel diameter had improved (Fig. 3B), but balloon dilation was performed for further dilation. The CA was observed to be dilated by balloon dilation while being observed by laparoscopy (Fig. 4C). IVUS scan confirmed that the diameter of the vessel had further improved (Fig. 3C). Total laparoscopic time was 3 h 32 min, total blood amount was 20 ml.

## Discussion

As treatment for intra-abdominal hemorrhage, IVR and damage control surgery are controversial. There is a report of mortality rates ranging from 12 to 50% in cases treated surgically for ruptured PDA aneurysms^8^. There is a report of hemostasis by IVR as the first choice for ruptured PDA aneurysms^4^. In our hospital, similarly, IVR is the first choice for the treatment of a ruptured PDA aneurysm if the diagnosis of PDA aneurysm is made preoperatively. IVR is performed by a cardiologist. If hemostasis fails, laparotomy is performed. We have had no IVR hemostasis failures in the past 5 years, and the reason for the success of IVR hemostasis in cases of ruptured PDA aneurysms in MALS is that the location of the aneurysm is the pancreatoduodenal arcade in all cases, which makes it easier to identify the site of bleeding^3^. Also, unlike traumatic hemorrhage, there is basically only one aneurysm to treat, which we believe contributes to the success rate of hemostasis with IVR. Conversely, if intra-abdominal bleeding is seen, MAL should be evaluated to ensure that there is no background MALS. In our hospital, retroperitoneal endoscopic MAL incision is performed for MALS as a curative procedure, though it has been reported that incision of the MAL is not always necessary^7^. However, in the case we report here, the second IVR confirmed stenosis of the CA. Without incision of MAL, the hemodynamics in the pancreatoduodenal arcade can become locally hypertensive, and there is a risk of aneurysmal reformation. MAL should be incised and the CA should be released. There is recent literature arguing for the necessity of MAL incision^9^, and in our hospital, MAL incision is planned after life-saving IVR for ruptured aneurysms. Treatment of MALS has been reported by laparotomic incision of the MAL, removal of the spinal plexus, and patch or bypass of the CA origin if stenosis persists^10^. Although the results of treatment with open surgery are also good, more and more reports have been published about laparoscopic surgery^5^,^6^. The retroperitoneal approach is attractive because there is few intraperitoneal manipulation and the risk of postoperative adhesive bowel obstruction is greatly reduced. A retrospective report about the outcomes of the retroperitoneal approach described the difficulty of CA identification^5^, and we also had difficulty in identifying CA. This is because there is few standard landmark for CA with retroperitoneal approach. Therefore, we performed simultaneous IVR to identify the CA and performed a MAL incision. Although this was the first case, the CA was easily identified. MAL incision with IVR is an effective procedure to identify CA. In our case, we can checked the expansion of the CA. Although each report states that contrast-enhanced CT is performed after surgery, there is little evidence that the MAL was definitely incised^2^–^7^. In our case, IVR and IVUS were performed before and after MAL incision. We also performed balloon dilation of the CA and were able to observe the dilation laparoscopically.

Although it is useful to be able to confirm that the MALS has been released, this is the first case and more cases need to be accumulated.

## Conclusion

A ruptured PDA aneurysm associated with MALS was embolized with IVR for life-saving purposes. Then retroperitoneal endoscopic MAL incision was performed with IVR. MAL identification by retroperitoneal approach had been difficult in the past, but IVR made it easy. Preoperative and postoperative dilation of the CA was also confirmed at the same time. This is a useful technique, and it is important to accumulate more cases to standardize the technique.

## Provenance and peer review

Not commissioned, externally peer-reviewed.

## Ethical approval

Ethical approval was not required.

## Consent

Written informed consent was obtained from the patient for publication of this case report and accompanying images. A copy of the written consent is available for review by the Editor-in-Chief of this journal on request.

## Sources of funding

None.

## Authors’ contribution

All authors namely were involved in the management of this patient. This manuscript has been drafted by all authors.

## Conflicts of interest disclosure

The authors declare that they have no financial conflict of interest with regard to the content of this report.

## Research registration unique identifying number (UIN)

None.

## Guarantor

Shoryu Takayama.
